# ﻿Palaearctic seed beetle *Bruchusaffinis* (Coleoptera, Chrysomelidae, Bruchinae) new to North America, arrival, distribution, and autecology

**DOI:** 10.3897/zookeys.1128.90016

**Published:** 2022-11-04

**Authors:** Hume B. Douglas, Stéphane Dumont, Karine Savard, Graham S. Thurston, Marilyn H. S. Light

**Affiliations:** 1 Canadian National Collection of Insects, Arachnids and Nematodes, Agriculture and Agri-Food Canada, Ottawa, ON, K1A 0C6, Canada Canadian National Collection of Insects, Arachnids and Nematodes Ottawa Canada; 2 Département de biologie et de biotechnologies, Collège Ahuntsic, 9155 rue Saint-Hubert, Montréal, H2M 1Y8, QC, Canada Département de biologie et de biotechnologies, Collège Ahuntsic Montréal Canada; 3 Canadian Food Inspection Agency, Ottawa Plant Laboratory, Building 18, 960 Carling Avenue, Ottawa, ON, K1A 0Y9, Canada Canadian Food Inspection Agency, Ottawa Plant Laboratory Ottawa Canada; 4 174, rue Jolicoeur, Gatineau, QC J8Z 1C9, Canada Unaffiliated Gatineau Canada

**Keywords:** Invasive alien species, biological control

## Abstract

First North American records are presented for *Bruchusaffinis* Frölich, 1799 (Coleoptera, Chrysomelidae, Bruchinae), as confirmed by morphology from multiple sites in Canada: British Columbia, Ontario, and Québec. Diagnostic information is presented for *B.affinis* in North America. This insect is expected to reduce plant reproductive output in infested *Lathyruslatifolius* L., *Lathyrussylvestris* L., and other potential *Lathyrus* (Fabaceae) hosts. Impacts on broad bean (*Viciafaba* L.) production are expected to be small. Potential reproductive impact on native North American *Lathyrus* species remains unknown. The United States of America and Canada are now known to be home to 69–79 species of adventive Chrysomelidae including 16–18 Bruchinae.

We have found two dead, teneral *B.affinis* individuals inside *Lathyrus* seeds imported from Europe, and we hypothesise that this species was introduced to Canada from Europe via seeds for planting sometime before 2007. At our study sites, *Lathyrus* flowering began in mid June followed by oviposition in late June with first adults emerging in late August, requiring about 60 days from egg to adult stage. *Dinarmusbasalis* (Rodani, 1877) (Hymenoptera, Pteromalidae) was newly recorded as parasitoid of *Bruchusaffinis* in Canada, and caused about 10% mortality in *B.affinis* at our sites.

## ﻿Introduction

The univoltine seed beetle *Bruchusaffinis* Frölich, 1799 is native to most countries of the western Palaearctic Region (Anton 2010). It has been found there as a seed predator of several species of *Lathyrus* and to some extent *Viciafaba* L, (broadbeans) (Bashar et al. 1987, 1990; Delobel and Delobel 2006). *Bruchusaffinis* adults use pollen and nectar of various *Lathyrus* species for food but require feeding on pollen of *L.latifolius* L. or *L.sylvestris* L. to terminate sexual diapause and commence oviposition on newly formed pods (Bashar et al. 1987, 1990). This species has not been observed to infest dried seeds (requirement for immature seeds is better documented for congener *Bruchuspisorum* Linnaeus, 1758; e.g. Howe and Currie 1964). So, although it damages seeds, *B.affinis* is not a pest of stored products.

**Figure 1.**
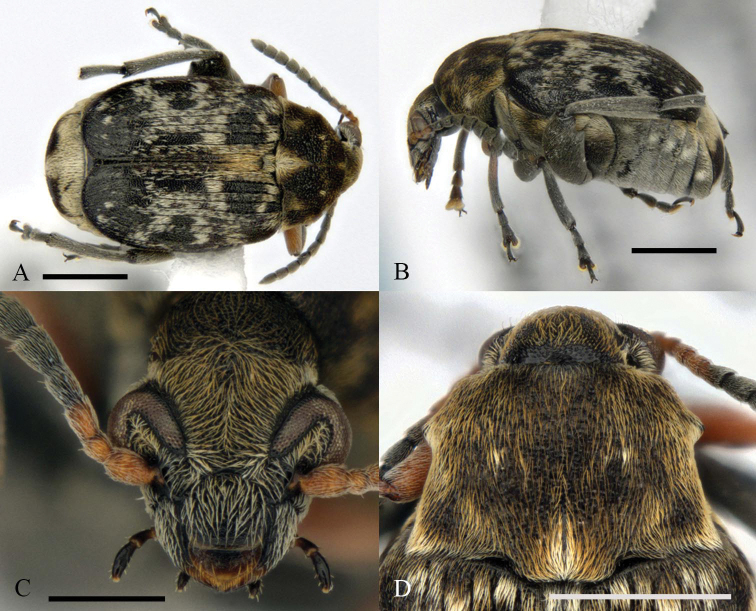
Morphology of a male of *Bruchusaffinis* from Québec, Canada **A** dorsal habitus **B** lateral habitus **C** anterior view of head **D** dorsal view of pronotum. Scale bars: 1 mm (**A, B, D**); 0.5 mm (**C**).

**Figure 2.**
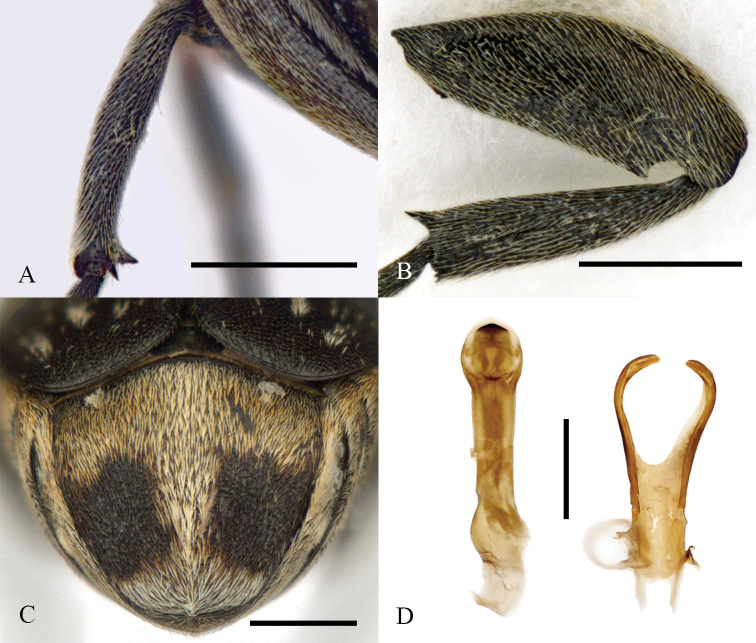
Morphology of a male of *Bruchusaffinis* from Québec, Canada **A** posterior view of mesotibia **B** lateral view of hind femur and tibia **C** pygidium **D** aedeagus. Scale bars: 0.5 mm.

*Lathyruslatifolius* (Fabaceae) is a perennial flowering vine introduced to North American gardens perhaps as early as the 1700s. It is now established as an ornamental and weedy species in Canada and USA. *Lathyrussylvestris* was introduced sometime before 1827 when seeds of both plants were listed in a Canadian nursery catalogue (Woodhead 1998). This study began in July 2020, when ML found the beetles (Figs 1, 2) emerging from seeds from the first pods of a solitary cultivated *L.latifolius* in Gatineau, Québec, Canada. This plant was started from seed in 2019 in a garden that otherwise did not contain *Lathyrus* plants. In 2021, ML found dead teneral *Bruchusaffinis* beetles within seeds inside a commercial packet of *L.latifolius* seeds imported from Europe. We aimed to investigate the range and invasion history of *Bruchusaffinis* first in that garden, and then more broadly in Canada.

We also examined the phenology and behaviour of *B.affinis* with *L.latifolius* and *L.sylvestris* in two Gatineau localities. This was to learn how *B.affinis* interacts with plant hosts in Canada. In particular, we examined the timing of flowering and the *B.affinis* egg to adult interval. We asked: at what date does *B.affinis* first appear on the hosts, mate, and begin oviposition? We also investigated the period required for eggs to hatch and for adult beetles to emerge from infested seeds.

## ﻿Methods

The first pods of *L.latifolius* to ripen in 2020 in the Gatineau garden represent the first infestation cohort for the site and plant (hereafter the index plant). Fifty-four seeds were harvested from that plant and dried at room temperature. Over the next few weeks, 18 beetles emerged, with a total seed infestation rate of 33%. Beetles were submitted to HD via the Agriculture and Agri-Food Canada Entomology National Identification Service. All specimens are deposited at the Canadian National Collection of Insects, Arachnids, and Nematodes (CNCI), 960 Carling Ave., Ottawa, Canada.

In 2021, all authors searched for additional populations of Bruchinae associated with *Lathyrus* spp. in Canada, examining growing plants, insect collections, and online photograph-sharing websites for specimens and observations. HD also contacted biologists with the Canadian Food Inspection Agency’s Plant Health Survey Unit to search *Lathyrus* plants for *Bruchus* beetles in other parts of Canada. Stephen Paiero checked the University of Guelph insect collection (Guelph, Ontario) for additional specimens of *B.affinis*, but found none. No comprehensive field survey has been conducted to determine the full range of this species in North America.

Beetle behaviour study locations were in Gatineau QC, a private garden, with only the *L.latifolius* index plant (45.464, −75.761). Additional beetles were observed in nearby roadsides, with eight patches of *L.sylvestris*, 45.4562, −75.7638 (2 m diameter); 45.4571, −75.7672 (3 m diameter); 45.4551, −75.7612 (3 m diameter); 45.455, −75.761 (2 m diameter); 45.4546, −75.7604 (5 m diameter); 45.4544, −75.7601 (1 m diameter); 45.4542, −75.7598 (6 m diameter); and 45.454, −75.7594 (2 m diameter). Historical weather data for Ottawa International Airport (Ontario, Canada) was obtained from Environment (Canada 2022).

### ﻿Plant material

**Figure 3.**
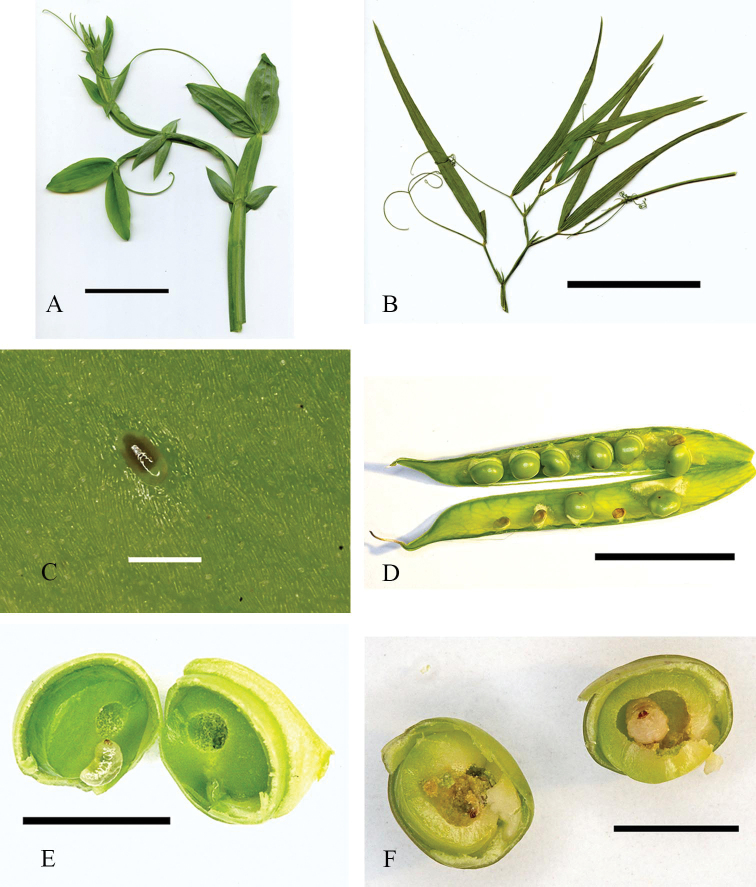
Morphology of *Lathyrus* spp. and *B.affinis* from Québec, Canada **A** apex of *L.latifolius* stem, garden plant (45.464, −75.561) **B***L.sylvestris* stem, pressed (45.457, −75.767) **C** egg of *B.affinis* on outer pod wall of *L.sylvestris***D** opened pod of *L.sylvestris* with seed entry holes of *B.affinis* larvae and white inner pod wall tissue developing beneath oviposition sites **E** infested seed of *L.sylvestris* with first instar larva of *B.affinis***F** same with second instar larvae. Scale bars: 50 mm (**A, B**); 0.5 mm (**C**); 25 mm (**D**); 5 mm (**E, F**).

Behavioural observations began with daily monitoring of the garden *L.latifolius* plant by ML for flowering and beetle activity from June through September 2021. First inflorescences having flowers open were tagged and then tracked. The first available pods were monitored for oviposition. Tagged pods remained on the plant until mature when they were harvested and permitted to dehisce. All remaining closed pods were opened manually two months after harvest. Seed infestation and parasitoid presence were assessed for all seeds from all tagged pods. Herbarium vouchers from both *Lathyrus* species were collected, for deposition in herbaria (CAN/DAO) (Fig. 3A, B).

Adult beetles were hand collected (i.e. not using a net) from *L.sylvestris* at behavioural study sites by ML in early morning. Specimens were preserved in 95% ethanol with several voucher specimens mounted. Stem tagging was not possible at the *L.sylvestris* roadside localities. So, randomly selected stems were used for study. Pods at varying developmental stages were examined and/or collected on several mornings from June to October. All seeds retained for rearing were stored at room temperature and examined daily for presence of emergent beetles. Maturing green to yellow seeds were opened within 24 hours of collection using a scalpel to view their contents (Fig. 5B). Mature brown seed samples, that floated when placed in water, were cracked open using a vacuum vise with exposed steel jaws. Seeds that sank in water and could not be cracked open were scored as uninfested. Additional pods were kept at 20 °C until January 2022 for further emergence of beetles or parasitoids.

Field photography was conducted by ML using an Olympus 12.5× Super Wide Optical Zoom camera. Macro photography was done using an Olympus EM5 MkII with macro lens according to subject and an Olympus T10 Ring Flash or an Olympus RFII Ring Flash for larger subjects. An Epson Perfection Scanner V550 Photo was used to document *Lathyrus* stems and herbarium specimens.

## ﻿Results and discussion

### ﻿Distributional records

The external morphology and male genitalia of Canadian specimens closely matched taxon concepts of *B.affinis* from Brandl (1981) and Borowiec (1988). A. Delobel, and L. Borowiec also confirmed our initial identifications based on submitted photographs. Specimens identified also matched Canadian National Collection specimens identified by Anton, Natterer, and Bottimer in morphology of the pronotum, male mesotibia, and aedeagus.

We found *B.affinis* at the following localities in **Canada: British Columbia**: Vancouver, 49.2162, −123.1713, 22.VI.2021, ex. L.latifolius, B. Spencer, 4 ex; **Québec**: Gatineau, 45.463, −75.765, reared from L.latifolius seeds from garden, M.H.S Light, 8 ex., CNC1053109 to CNC1053117; Gatineau, 5.IX.2021, M.H.S. Light; Wakefield, 45.6466, −75.9298, reared from L.sylvestris seeds of field edge patch,15.VIII.2021, M.H.S. Light, 12 ex.; Gatineau, boul. Cité des Jeunes, 45.4544, −75.7601, mating on flowering L.sylvestris of roadside patch, 27.VI.2021, M.H.S. Light, 5 ex.; Gatineau, 45.4546, −75.7604, 27.VI.2021, M.H.S. Light, 18 ex.; Gatineau, 45.464, −75.761, reared from L.latifolius seeds from garden, 18.VIII.2021, M.H.S. Light, 1 ex.; Gatineau, 45.71, −75.7672, reared from L.sylvestris seeds of a roadside patch, 15.VIII.2021, M.H.S. Light, 5 ex.; Gatineau, 45.4551, −75.7612, reared from L.sylvestris seeds, 15.VIII.2021, M.H.S. Light, 8 ex.; Gatineau, 45.464, −75.761, infesting L.latifolius seeds; Gatineau QC, eggs on pod 25.VI.2021, eggs hatch 6.VII, beetles hatch 18.VIII.2021, M.H.S. Light, 8 ex.; Gatineau, 45.464, −75.761, Dead Bruchusaffinis from two seeds in a sealed commercial package of 25 L.latifolius seeds, 18.21.III.2021, M.H.S. Light, 2 ex.; Farm Point, 45.6092, −75.8975, 23.VI.2021, ex. roadside Lathyrus flowers, H. Douglas, 6 ex.; Laval, Boisé Papineau (Près St. Martin) sur marguerite, 45.60229, −73.68096, 19.VI.2013, S. Dumont, 1 ex.; Laval, Boisé Papineau, 45.60704, −73.68082, battage spirée en fleurs, 21.V.2014, S. Dumont, 1 ex.; Laval, Boisé Papineau, 45.603, −73.681, sur *Lathyrus*, 29.VII.2014, S. Dumont, 3 ex.; Laval, Contrecoeur, Camp des Grèves 45.979, −73.182, sur *Lathyrus*, 7.VIII.2009, S. Dumont, 3 ex.; Laval, Berge Olivier-Charbonneau 45.698, −73.529, sur *L.latifolius*, 21.VII.2021, S. Dumont, 22 ex.; Montréal, Anjou, près Parc Roger Rousseau 45.617, −73.545, sur *L.latifolius*, 22.VII.2021, S. Dumont, 20 ex.; Notre-Dame-de-l’île-Perrot (Vaudreuil), 4.VII.2007, Battage *Lathyrus* sp., Pierre de Tonnancour, 2 ex.; Notre-Dame-de-l’île-Perrot (Vaudreuil), 20.V.2011, Battage *Caragana* sp., Pierre de Tonnancour, 1 ex.; Notre-Dame-de-l’île-Perrot (Vaudreuil), 14.VI.2011, Battage *Cirsiumarvense*, Pierre de Tonnancour, 1 ex.; Notre-Dame-de-l’île-Perrot (Vaudreuil), 20.V.2011, Battage *Rubusodoratus*, Pierre de Tonnancour, 1 ex.; Notre-Dame-de-l’île-Perrot (Vaudreuil), 31.VIII.2011, Battage *Lathyruslatifolius*, Pierre de Tonnancour, 1 ex.; Notre-Dame-de-l’île-Perrot (45.3775, −73.9431), 7.VI.2019, fauchage, champ humide, Pierre de Tonnancour, 1 ex. **Ontario**: Ottawa, Hurdman Bridge, 45.418, −75.664, 3.VII.2021, ex. *Lathyrus* flowers, 8 ex., H. Douglas (field photographs: https://www.inaturalist.org/observations/85903171. An additional female *Bruchus* specimen from near Québec City, seen only in internet photographs also has pronotal morphology matching *B.affinis* (https://www.inaturalist.org/observations/38795458), suggesting that *B.affinis* may also be established in that region.

We present evidence of 79 specimens from multiple sites in three provinces, separated by over 3500 km over 14 years, and rearing evidence of successful reproduction in nature. Together, these lead us to conclude that multiple populations of *B.affinis* are established in Canada.

*Bruchusaffinis* can be distinguished from other North American *Bruchus* by the following combination of characters (adapted from key by Kingsolver 2004 using Borowiec 1988): antennae with three or four basal antennomeres red-brown; pronotum with lateral spines situated before pronotal midlength in anterodorsal view (Fig. 1D); male mid tibiae with two spines on ventral surface near apex (Fig. 2A); hind legs with femoral spine (Fig. 2B) extending apicad (not protruding ventrad beyond basal part of ventral surface, like Kingsolver 2004 fig. 257), mucro twice longer than lateral denticle (Fig. 2B); protibiae with some red-brown colouration on basal half, mesotibiae black (Fig. 1B). The broadly obtuse apex of the aedeagus is also unique among North American species (Fig. 2D). For rapid assessments, no other species has lateral spines situated on the anterior half of the pronotum and all black mesotibiae.

### ﻿Adventive species biology

It was initially unknown whether the infestation of the index plant could represent the first establishment of a new population of *B.affinis* in Canada. Alternatively, we thought this infestation could have spread from a pre-existing adventive population. Later we found nearby patches of *B.affinis*-infested *L.sylvestris*. Finding these specimens as well as others from elsewhere in Canada (many collected before 2020) is consistent with spread of a pre-established adventive population rather than establishment of a new adventive population in the garden of the index plant resulting from planting infested seeds there.

Our finding of *B.affinis* specimens within seeds of *L.latifolius* that were commercially imported from Europe suggests that import of infested seed is a likely pathway for the introduction of this species to Canada. The discovery of *B.affinis* in Québec, Ontario, and British Columbia over only 15 years is consistent with multiple introductions over several years since our earliest specimen collection date in 2007. This is also consistent with movement of infested seed rather than haphazard introduction of adults (e.g. live beetles accidentally trapped in shipping containers). This evidence indicates that *B.affinis* was present in Canada for 15 or more years.

*Bruchusaffinis* is known to infest seed pods of *L.grandiflorus*, Sibth. & Smith, *L.latifolius*, *L.sylvestris*, and *L.tuberosus* L. (Kergoat et al. 2007), all introduced species to North America, and *Vicia* sp. (Fabres et al. 1986). Knowledge that *B.affinis* harms only seeds indicates that it will not harm the ability of these plants to grow or perform ornamental and erosion control functions. The moderate observed impact of *Bruchus* predation on their seeds may somewhat reduce these plants’ ability to propagate. USA and Canada are also home to 29 named native *Lathyrus* species (USDA, 2022) plus other native Fabaceae. It remains unknown whether *B.affinis* can harm the reproductive outputs of these native species.

*Bruchusaffinis* is also known to use *Viciafaba* as host in Europe (Segers et al. 2021), where it appears to act as a minor pest. In Europe, several species of *Bruchus* infest fava beans in the field. Of these, *B.rufimanus* is the most destructive (Segers et al. 2021). *Bruchusrufimanus* is listed as present in Canadian provinces producing fava beans (Bousquet et al. 2013); however, we were unable to find evidence that it causes economic damage to Canadian fava bean crops (e.g. Agrinova 2017). Together, these two findings may suggest that *B.affinis* is unlikely to cause substantial harm to fava beans in Canada.

This new North American record, added to the species counts by Douglas et al. (2021), indicate that Canada and the USA are together known to host 69–79 species of adventive Chrysomelidae, including 16–18 Bruchinae. Of these, 52–60 adventive Chrysomelidae are known from Canada, and 56–66 are known from the USA.

### ﻿Behavioural observations

Flowering of both *Lathyrus* species began the week of June 15, 2021. The first *L.latifolius* pods developed by June 23 with the first eggs appearing on pods June 25: hatching began July 6. Mature, undehisced pods were collected from August 2 to August 12. Twenty-one *B.affinis* emerged from the 76 seeds (22 pods) on August 18–26 only: there were no further emergences. Seed infestation rate was 27.6%, averaging about one *B.affinis* larva per pod. Of the 21 tagged pods, 20 contained eggs (range 1–8 eggs per pod; median 3 eggs per pod). Overall, beetle reproduction on *L.latifolius* commenced as soon as flowers developed. Development of the new beetle generation was complete by the end of August, with about one quarter of seeds killed by beetles.

On *L.sylvestris*, 30 *B.affinis* adults were observed on June 27 on plants at five of eight roadside patches. Few *B.affinis* were seen after decrease in flower production in mid-July. The first 43 *B.affinis* individuals reared indoors emerged on August 14. Emergence of *B.affinis* collected from all eight patches on July 28 (104 pods/606 seeds) occurred mainly during two peak periods with warm weather: August 14–21 (preceded by five days of hot weather, 30–31 °C; indoor 27 °C), 68 beetles; and August 25–26 (daily max. 32.8–32.7 °C; indoor 27 °C; 92 beetles). Of all 215 beetles eclosed in August 2021, 160 were from these two periods. The infestation rate of the 606 seeds was 35% (13.5–73.5% across patches). If a pod had not yet dehisced, emerging beetles chewed exit holes to exit through the pod wall. The sound of chewing was audible outdoors from up to 2 m away. All observed beetles initiated flight immediately after exiting pods outdoors or within rearing containers. Flying behaviour in containers continued for several hours, after which emerging beetles no longer attempted flight. Dehisced or manually opened pods of both species revealed white tissue developing from the inner pod wall beneath egg positions (Fig. 3D). Beetle activity periods and seed infestation rates on *L.sylvestris* were similar to those on garden grown *L.latifolius*.

**Figure 4.**
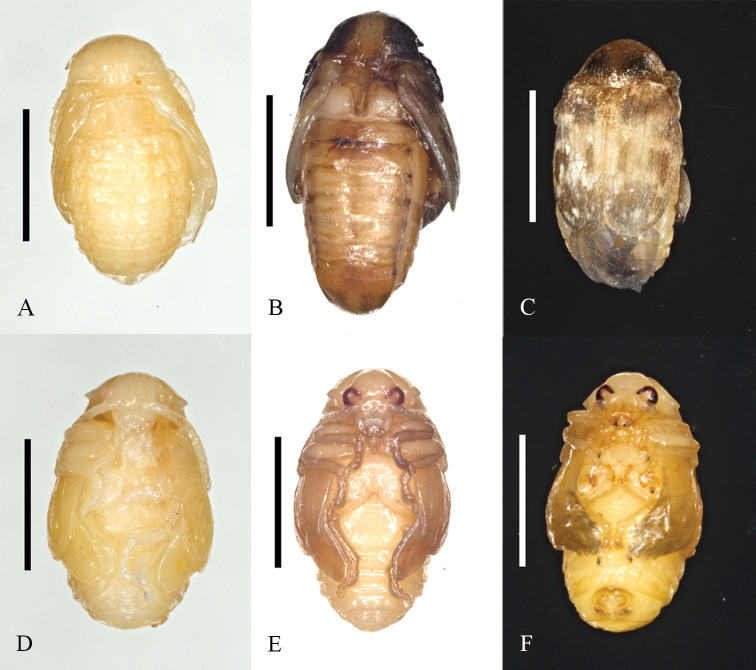
*Bruchusaffinis* pupal stages in seeds of *L.sylvestris* from Québec, Canada. Scale bars: 2 mm.

**Figure 5.**
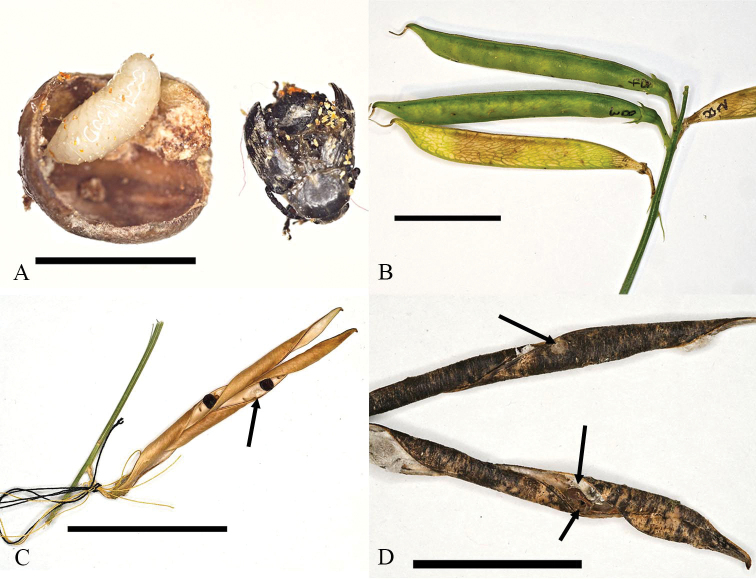
**A***Bruchusaffinis*, parasitized by Hymenoptera larvae in seed of *L.sylvestris***B** maturing pods of *L.sylvestris* with hatched and unhatched eggs **C** dehisced pod of *L.latifolius***D** dehisced pod of *L.sylvestris* showing *B.affinis* exit holes in pod wall and seed. Scale bars: 2 mm (**A**); 25 mm (**B**); 50 mm (**C**); 20 mm (**D**).

The earliest evidence of parasitism was September 1 when two different hymenopteran larvae were found within *B.affinis*-infested seeds. Hymenoptera parasitoids of *B.affinis* first emerged from mature seeds of *L.sylvestris* (collected September 5, 10, and 11. These were identified as *Dinarmusbasalis* (Rodani, 1877), a cosmopolitan parasitoid of Bruchinae in legume seeds (Schmale et al. 2003) by John Huber and Gary Gibson (CNCI). *Bruchusaffinis* and parasitoid emergence holes from seeds were distinguishable, allowing ML to score beetle and parasitoid infestation rates. Holes made by reared *B.affinis* were circular, 1.5–2.1 ± 0.02 mm in diameter (*n* = 63, median: 1.9 mm), while those from parasitoids were smaller and circular: 0.6–1.2 ± 0.02 mm (*n* = 27, 0.8 mm). The 438 seeds collected for rearing from patch 5 on September 5 resulted in 19 parasitoid emergence holes plus one pre-emergent parasitoid or 8% parasitism of developing *B.affinis*. *Bruchusaffinis* was confirmed as host of the parasitoid by examination of the host remains by ML. In most cases, the remains of the beetle, including elytra and mandibles, could be used to confirm that a pupa (Fig. 4) was host to the parasitoid. Fig. 5A shows a parasitoid larva was feeding on a teneral beetle when a field-collected seed was opened on October 26. Here, the parasitoid may have continued feeding later into the autumn because of warmer indoor rearing conditions.

### ﻿Reproductive phenology

Onset of host flowering was about two weeks earlier in Québec (mid-June) than reported from France (early July) (Bashar et al. 1987). Fabres et al. (1986) reported that adult *B.affinis* in France lived about 11 months after emergence including a nine-month reproductive diapause reporting that first pods appropriate to oviposition were present in mid-July with a maximum number of available pods for oviposition present in mid-August. We found that eggs were laid at our study site slightly earlier (about June 25). The period of sexual reproduction of *B.affinis* was short. We observed few *B.affinis* on *Lathyrus* plants after mid-July.

In summary, we report the following from North America (Québec): infestation of *L.latifolius* and *L.sylvestris* (Fabaceae) by *Bruchusaffinis*; rearing of *B.affinis* from both hosts; oviposition in 2021 beginning late June, to adult emergence beginning mid August; observation of *B.affinis* adults chewing holes to exit undehisced pods of *L.sylvestris*; parasitization of *B.affinis* by *Dinarmusbasalis* in Québec. We also found that the seed emergence hole diameter of *B.affinis* was consistently larger than that of parasitoid emergence holes, such that we could reliably distinguish between exit hole types. We quantified *B.affinis* seed infestation rates of *L.latifolius* (17–28%), and of *L.sylvestris* (13.5–73.5%) for 2021.

## ﻿Conclusions

*Bruchusaffinis* is established in North America in Canada in Québec, Ontario, and British Columbia since 2007 or earlier using introduced *Lathyruslatifolius* and *L.sylvestris* as hosts. These populations may have originated from imports of infested seed for planting. Numbers of recorded adventive Chrysomelidae for Canada and America north of Mexico are updated to reflect this finding. Beetle development involved an approximately two-month period from oviposition to emergence. Phenological milestones of adult flight activity, oviposition, and adult emergence were estimated for study populations. Parasitoids were documented to use *B.affinis* as a host in Canada, but had a minor impact on infestations, accounting for about 10% mortality of pre-emergent pupae.
